# In situ repair of segmental loss posterior lateral meniscal root tears outperforms meniscofemoral ligament imbrication in the ACL reconstructed knee

**DOI:** 10.1186/s40634-023-00572-5

**Published:** 2023-01-26

**Authors:** Max Seiter, Brenton W. Douglass, Alex W. Brady, Grant J. Dornan, Justin R. Brown, Thomas R. Hackett

**Affiliations:** Steadman Philippon Research Institute, Vail, CO USA

**Keywords:** Meniscofemoral ligament imbrication, ACL reconstruction, Segmental loss posterior lateral meniscal root tears

## Abstract

**Purpose:**

The purpose of this study was to compare the biomechanical effect of in-situ repair of posterior lateral meniscal root (PLMR) tear with segmental meniscal loss, with and without meniscofemoral ligament (MFL) imbrication, on anterior cruciate ligament (ACL) graft force and knee joint kinematics.

**Methods:**

Ten fresh-frozen cadaveric knee specimens underwent kinematic evaluation in five states: 1) Native, 2) ACLR, 3) Segmental PLMR loss, 4) In-situ PLMR repair, and 5) MFL augmentation. Kinematic evaluation consisted of five tests, each performed at full extension and at 30° of flexion: 1) Anterior drawer, 2) Internal Rotation, 3) External Rotation, 4) Varus, and 5) Valgus. Additionally, a simulated pivot shift test was performed. Knee kinematics and ACL graft force were measured.

**Results:**

PLMR tear did not significantly increase ACL graft force in any test. However, PLMR repair significantly reduced ACL graft force compared to the ACLR alone (over constraint -26.6 N, *p* = 0.001). PLMR tear significantly increased ATT during the pivot shift test (+ 2.7 mm, *p* = 0.0001), and PLMR repair restored native laxity. MFL augmentation did not improve the mechanics.

**Conclusions:**

In-situ PLMR repair eliminated pivot shift laxity through ATT and reduced force on the ACL graft, indicating that this procedure may be ACL graft-protective. MFL augmentation was not shown to have any effect on graft force or knee kinematics and untreated PLMR tears may place an ACL graft at higher risk. This study suggests concomitant repair to minimize additional forces on the ACL graft.

## Background

Posterior lateral meniscal root (PLMR) tears are commonly encountered in patients who have sustained injury to the anterior cruciate ligament (ACL) with an incidence between 6.6–14% [2, 3, 6, 11, 19, 25]. PLMR tears are rare in the absence of ACL tear and are 10.3 times more likely to occur with an ACL injury than posterior medial meniscal root tears [3, 14, 26]. In addition, PLMR tears also frequently present in combination with lateral femoral condyle bone bruises and tibial plateau bone bruises, a constellation of injury that has been coined “lateral quartet” [6, 10].

The PLMR has an important contribution to knee laxity, particularly as a ﻿secondary stabilizer to rotational torque, e.g., during a pivoting maneuver [8, 15, 16, 23]. It has been hypothesized that untreated PLMR tears are a cause for ACL graft failure [16, 17]. This failure may be due to increased anterior tibial translation, increased rotatory laxity, and increased force on the graft [8, 24]. In a biomechanical investigation of anatomic repair of PLMR tears without segmental meniscal loss in conjunction with ACL reconstruction, repair was shown to reduce anterior tibial translation and ACL graft force [24]. However, segmental loss of the PLMR and subsequent in-situ repair was not evaluated. Frequently, the PLMR tears seen with concomitant ACL injuries are LaPrade type 2 tears [12, 26]. Type 2 meniscal root tears are complete radial tears within 10 mm of the root attachment, and may be associated with segmental meniscal loss, particularly in type 2C tears, which are located 6-9 mm from the root attachment [12].

Additionally, it has been observed that PLMR tears with an intact meniscofemoral ligament (MFL) do not exhibit the same degree of meniscal extrusion as those without an intact MFL [2, 11, 20]. Some authors have suggested that meniscal function is preserved in PLMR tears with an intact MFL, as measured by contact pressures in the lateral compartment; and have advocated that these tears may not require treatment [1, 4, 5, 9, 22]. The idea of testing these relationships has yet to be elucidated and if successful, this study would provide another tool for surgeons to more effectively manage ligamentous knee joint damage.

The purpose of this study was to compare the biomechanical effect of in-situ repair of PLMR tear with segmental meniscal loss, with and without MFL imbrication, on ACL graft force and knee joint kinematics. It was hypothesized that in-situ PLMR repair and MFL augmentation would decrease anterior translation, internal/external rotation, and ACL graft force compared to segmental PLMR loss in all tests.

## Material and methods

### Specimen preparation

**Fig. 1:**
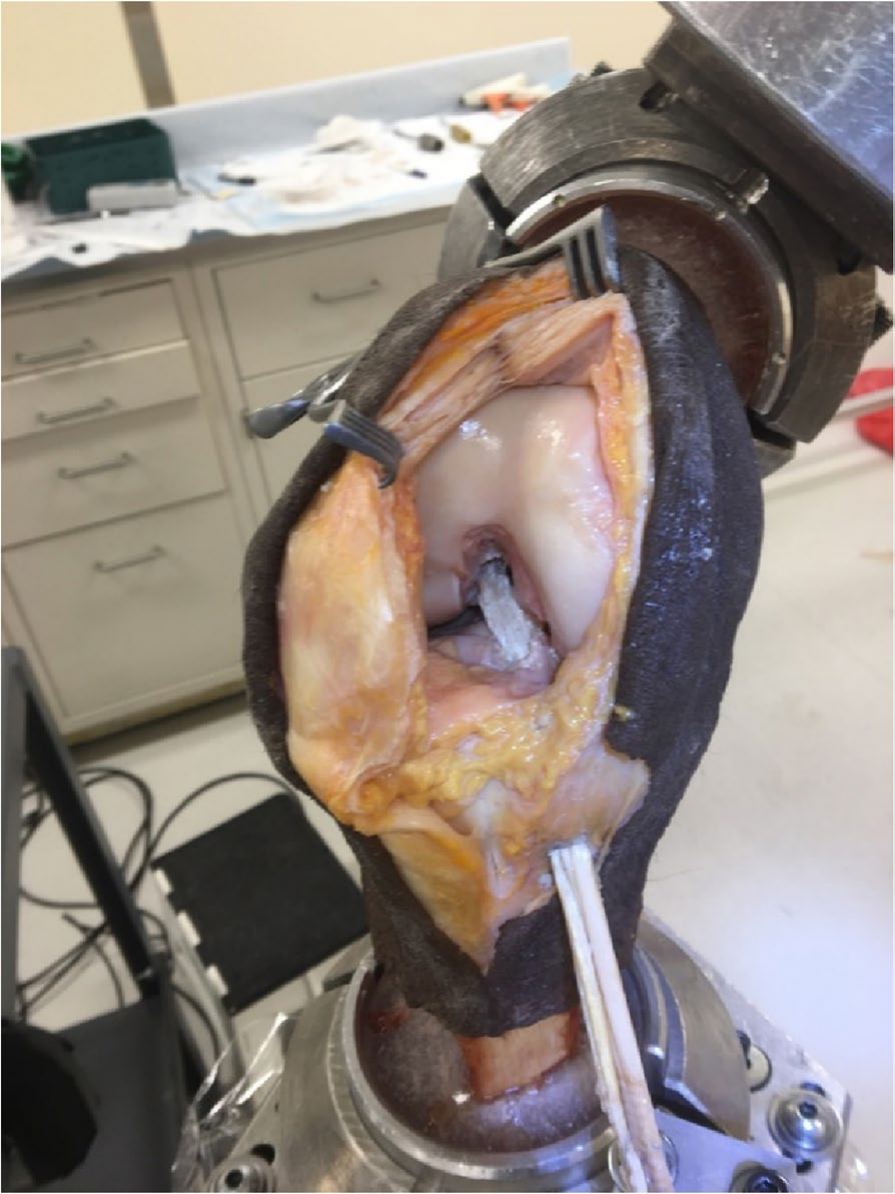
Quadrupled hamstring graft after fixation with femoral cortical suspensory button

Institutional review board approval was not required because de-identified cadaveric specimens are exempt from review at our institution. Ten fresh-frozen, non-paired, cadaveric knee specimens including 20 cm of femur and 20 cm of tibia were dissected of all skin and subcutaneous. Specimens were stored at -20 °C and thawed at room temperature for 24 h prior to preparation and testing. Inclusion criteria were 65 years or younger, a BMI less than 35 kg/m^2^, and no prior history of injury, surgery, or degenerative joint disease. All specimens were screened arthroscopically and excluded if ligamentous, meniscal, or cartilage pathology (beyond Outerbridge grade one) existed. The fibula and tibia were potted in polymethyl methacrylate (PMMA; Fricke Dental International, Streamwood, IL) in a custom cylindrical mold with the joint line oriented parallel to the potting surface, and the femur was similarly potted (Fig. 1).

### Surgical technique

**Fig. 2:**
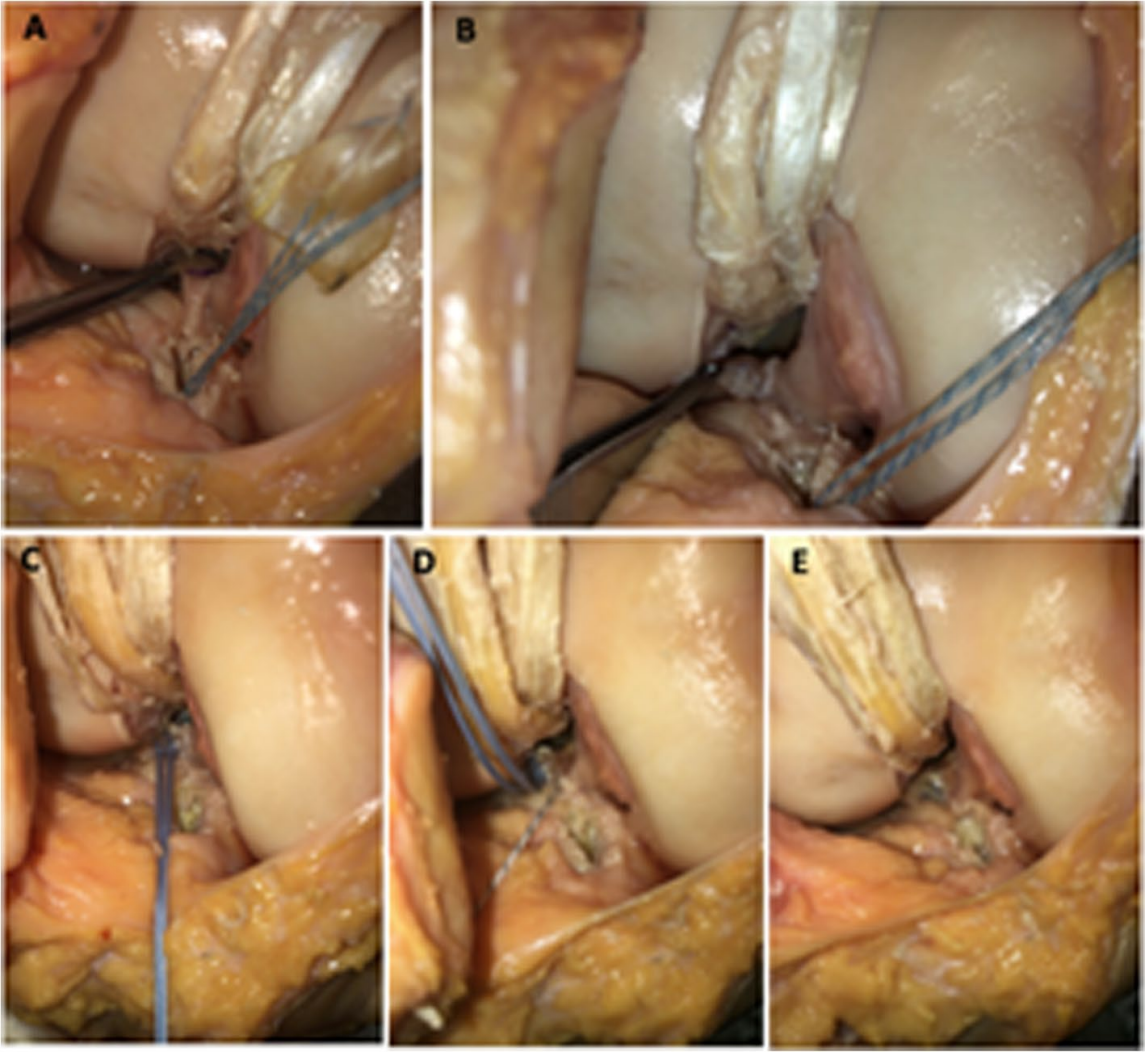
**A** Posterior lateral meniscal root marked at 8 mm from the center of its attachment site. **B** PLMR with segmental meniscal root loss and intact MFL. **C** PLMR with two meniscal repair stitches passed in luggage-tag fashion. **D** MFL with an imbrication stitch passed in luggage-tag fashion. **E** Completed PLMR root in-situ repair and augmentation with MFL imbrication

To create the simulated ACL injury, a medial parapatellar arthrotomy was performed, and the ACL was resected using a scalpel. The ACL was then reconstructed using a quadrupled hamstring allograft and anatomic single bundle, transtibial-technique. Grafts were whipstitched in the standard fashion with a #2 suture (FiberWire, Arthrex; Naples, FL), and sized to 10 mm. The tibial tunnel was drilled with a standard point-to-point ACL guide set to 55 degrees, (Arthrex; Naples, FL). The femoral tunnel was drilled with a 7 mm offset guide loaded with a 2.4 mm spade tip pin (Arthrex; Naples, FL). Each were opened with a 10 mm reamer, the lateral femoral cortex was preserved with 7 mm of lateral bone. The ACL graft was passed through the tibial tunnel, and into the femoral tunnel. The cortical suspensory fixation device was flipped such that it was firmly on the lateral cortex and tensioned to firmly seat the graft on the femoral side. The tibial end of the graft was secured to the S load cell, as shown on Fig. 2. The joint was then sealed with a wrap to simulate joint capsule closure.

To create the simulated PMLR tear, the posterior horn of the lateral meniscus was transected with a scalpel at a distance measured 8 mm from the root attachment. This tissue between was then completely excised, creating the segmental defect: a complete radial tear 8 mm from the root, with tissue loss involving the meniscal root. The meniscofemoral ligament was left intact, as shown in Fig. 2A and B.

The PLMR tear repair was performed using two-tunnel tibial pull-through technique with two sutures in luggage tag configuration (FiberLink and TigerLink SutureTape, Arthrex; Naples, FL), tied over a suture button to the face of the tibia [7]. A self-retaining suture passer (Firstpass Mini, Smith and Nephew, Andover MA), was used to pass the luggage tag sutures through the meniscal root remnant (Fig. 2C). Similarly, for the MFL augmented state, the posterior MFL was captured with a luggage tag suture by the use of the self-retaining suture passer (Fig. 2D). The sutures were tensioned to assess the extent of medial excursion of the PLMR remnant towards the anatomic footprint, and tunnels were drilled at the point of maximal excursion. Two tibial tunnels approximately 5 mm apart were drilled with the use of a meniscal root guide and cannulated guide pins (Acufex, Smith and Nephew, Andover MA). Finally, the sutures were pulled though the tibia by use of a passing stitch and were tied over the bone bridge with a cortical button.

For the MFL augmentation state, the MFL stitch was passed through the more anterior and medial tibial tunnel and was tied over the tibial button (Fig. 2E).

### Robotic testing setup

The potted tibia was securely clamped to a custom fixture mounted onto a universal force/torque sensor (Delta F/T Transducer; ATI Industrial Automation), set on a stationary pedestal. The tibia fixture was equipped with an S load cell (model 60,050–100; Vishay Intertechnology) mounted to a pivoting attachment point and fitted with a custom soft tissue clamp to measure the tension force in line with our sensor termed ACL graft force for this study. The potted femur was secured to a similar fixture attached to the end effector of the six degrees of freedom robotic arm (KUKA KR 60–3, KUKA Robotics, Augsburg, Germany). Figure 1 shows the full robotic testing setup. A coordinate measuring machine (Romer Absolute Arm, Hexagon Metrology, North Kingstown, RI) was used to collect twelve points on each knee: 1) medial femoral epicondyle, 2) lateral femoral epicondyle, 3) medial tibial plateau, 4) lateral tibial plateau, 5–8) four points forming a ring around the distal tibia, 9–12) four points forming a ring around the proximal femur. These points were used to establish a tibia, femur, and joint coordinate frame, as described in the SimVITRO software (SimVITRO, Cleveland OH, 44,106).

### Biomechanical testing protocol

Each knee underwent kinematic testing by the Kuka robot in the following states: 1) Intact, 2) ACL reconstruction, 3) Segmental lateral meniscal root tear, 4) Lateral meniscal root repair, and 5) Lateral meniscal root repair augmented with meniscofemoral ligament imbrication. The order of states 4 and 5 was randomized, such that each procedure was performed first on half the specimens. ROM was tested on all specimens to ensure capability to perform all desired movements for the study. The ACL was re-tensioned to 88 N in full extension between each state after the ACL reconstruction state for consistency between states. Kinematic evaluation consisted of a total of eleven tests, performed in a randomized order. Five tests were performed at full extension and at 30° of knee flexion: 1) 88-N Anterior drawer, 2) 5-Nm Internal Rotation (IR), 3) 5-Nm External Rotation (ER), 4) 5-Nm Varus, and 5) 5-Nm Valgus. Additionally, a simulated pivot shift test, consisting of combined 5-Nm internal rotation, 5-Nm varus, and 88-N anterior load, was run at 30° of flexion [8]. The order of the tests was randomized to avoid confounding findings from one sequence of motion. All tests were performed at a fixed flexion angle, and a 20-N compressive load was applied to seat the joint. The forces and torques on the other axes were set to 0 N and 0 Nm, respectively. Tests were programmed to end when all forces were within 2 N of their targets and all torques were within 0.2 Nm of their targets for five consecutive seconds. Anterior tibial translation (ATT) was measured by the Kuka robot and reported in mm for the anterior drawer and simulated pivot shift tests, and knee range of motion was reported in degrees for the IR, ER, varus, and valgus tests. ACL Graft force was recorded during all tests.

### Statistical analysis

Random-intercepts linear mixed-effects models were used to compare experimental conditions during each of 11 simulated exams while accounting for the repeated measures nature of the study design. Estimated marginal means were reported and Tukey’s method was used to make all pairwise comparisons among the knee states. Residual diagnostics were inspected to ensure model fit and that assumptions were met. Tukey adjusted *p*-values less that 0.05 were considered statistically significant. The statistical software R version 4.0.0 was used for all plots and analyses (access date June 18, 2020; R Core Team, Vienna Austria; with additional packages nlme and emmeans).

## Results

**Fig. 3:**
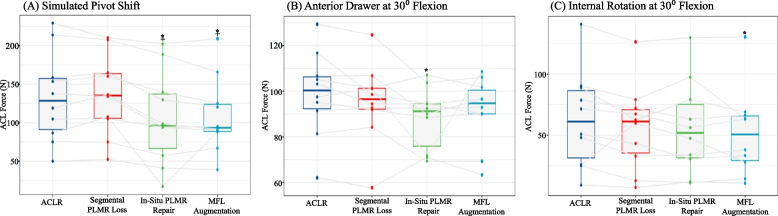
Box plot graphs demonstrating median force in newtons for (**A**) the simulated pivot shift, (**B**) the anterior drawer at 30° flexion and (**C**) the internal rotation at 30° flexion tests. Dots represent individual specimen observations. Thick horizontal lines represent group medians, while top and bottom of boxes represent the 25^th^ and 75^th^ percentiles, respectively. *: Significantly different from ACLR, + : Significantly different from Segmental Meniscus

**Fig. 4:**
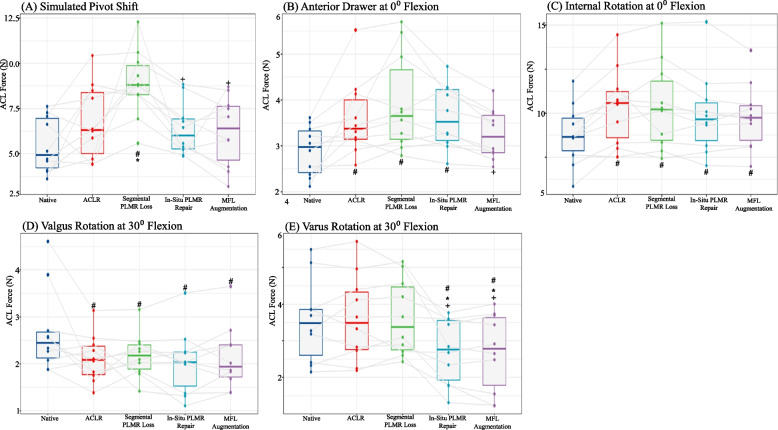
Box plot graphs demonstrating median anterior tibial translation in mm for (**A**) the simulated pivot shift and (**B**) the anterior drawer at 0° flexion tests, and range of motion in ° for (**C**) the internal rotation at 0° flexion, (**D**) valgus rotation at 30° flexion and (**E**) varus rotation at 30° flexion tests. Dots represent individual specimen observations. Thick horizontal lines represent group medians, while top and bottom of boxes represent the 25^th^ and 75^th^ percentiles, respectively. #: significant from native; *: significant from ACLR; + : significant from segmental meniscus

Tables 1, 2 and 3 show the mean ACL graft force, anterior tibial translation, and range of motion results for all tests, respectively. For tests showing at least one significant difference between states, box plots were created (Figs. 3 and 4).

**Table 1:** Mean ACL Graft force (N) by State (columns) and Test (rows)

Test\State	ACLR	Segmental PLMR Loss	In-Situ PLMR repair	MFL Imbrication
Simulated Pivot Shift	132.8 ± 58	135.3 ± 52.3	106.2 ± 60.2	60.2109 ± 49
AD0	108.9 ± 13.6	104.2 ± 11.3	106.8 ± 10.6	10.6109.7 ± 13.7
AD30	98.9 ± 18.6	95.4 ± 17.1	88.3 ± 13.4	13.491.5 ± 14.7
ER0	44.7 ± 23.7	35.1 ± 18.5	43.9 ± 24.1	24.137.7 ± 14.7
ER 30	16.9 ± 16.1	12.7 ± 10.5	14.1 ± 10.8	10.815.6 ± 14.6
IR 0	92.2 ± 31.9	90.2 ± 25.6	94.3 ± 26.5	26.592.1 ± 28.1
IR 30	62.9 ± 39.2	56.4 ± 34.8	56.1 ± 37.9	37.951.7 ± 35.2
Valgus 0	36.4 ± 17	29.1 ± 10.4	34.8 ± 14.1	14.131.7 ± 9.3
Valgus 30	17.2 ± 15.8	20.4 ± 11.6	13.6 ± 6.4	6.413.7 ± 7.8
Varus 0	52.4 ± 12.5	49.5 ± 11	47.5 ± 20.1	20.151.3 ± 12.7
Varus 30	25.8 ± 19.5	22.8 ± 15.3	21.9 ± 14.2	14.222.8 ± 13.2

**Table 2:** Mean ATT (mm) by State (columns) and Test (rows)

Test\State	ACLR	Segmental PLMR Loss	In-Situ PLMR repair	MFL Imbrication
Simulated Pivot Shift	5.4 ± 1.2	6.4 ± 1.7	8.1 ± 1.5	1.56.1 ± 1.2
AD 0	2.9 ± 0.5	3.6 ± 0.8	3.9 ± 1.1	1.13.6 ± 0.7
AD 30	5.4 ± 1.9	6.6 ± 1.9	6.9 ± 1.7	1.76.9 ± 2.6

**Table 3:** Mean ROM (degrees) by State (columns) and Test (rows)

Test\State	ACLR	Segmental PLMR Loss	In-Situ PLMR repair	MFL Imbrication
ER 0	9.6 ± 2.4	9.1 ± 2	9.4 ± 2.3	2.39.5 ± 2.3
ER 30	18 ± 2.4	17.4 ± 5.4	17.4 ± 5.5	5.518.5 ± 6.4
IR 0	8.8 ± 1.7	10.4 ± 2.2	10.4 ± 2.5	2.59.9 ± 2.3
IR 30	18.6 ± 2.9	18.9 ± 6.3	16 ± 4.8	4.818 ± 6.2
Valgus 0	1.1 ± 0.4	1.1 ± 0.5	1.1 ± 0.5	0.51 ± 0.4
Valgus 30	2.7 ± 0.9	2.1 ± 0.5	2.2 ± 0.5	0.52 ± 0.7
Varus 0	1.1 ± 0.5	1.2 ± 0.5	1.1 ± 0.5	0.51.1 ± 0.7
Varus 30	3.5 ± 1.1	3.6 ± 1.2	3.6 ± 1	12.7 ± 0.9

### ACL graft force

ACL graft force was reported in all robotic tests, and 3/11 tests showed significant differences between states: pivot-shift (Fig. 3A), anterior drawer at 30° of knee flexion (Fig. 3B), and internal rotation at 30° of knee flexion (Fig. 3C).

### ACL graft force – simulated pivot shift

During pivot shift testing, there were no significant differences in ACL graft force between the “ACLR” and “segmental meniscus loss” states (*p* = 0.977), or between the “Meniscus Repair” and “MFL augmentation” states (*p* = 0.968). However, there was a significant decrease in ACL graft force in the “Meniscus Repair” state compared to both the “ACLR” state: (-26.6 N, *p* = 0.001) and the “segmental meniscus loss” state: (-29.1 N, *p* < 0.01). This decrease in ACL graft force held true in the “MFL augmentation” state compared to the “ACLR” state: (-23.8 N, *p* = 0.004) and the “segmental meniscus loss” state: (-26.3 N, *p* = 0.01).

### ACL graft force – anterior drawer at 30 degrees of flexion

During anterior drawer testing at 30° of knee flexion, there was a significant decrease in ACL graft force in the “Meniscus repair” state compared to the “ACLR” state: (-10.6 N, *p* = 0.026). No further statistically significant differences were observed between states in this test.

### ACL graft force – internal rotation at 30 degrees of flexion

During internal rotation testing at 30° of knee flexion there was a significant decrease in ACL graft force in the “MFL augmentation” state compared to the “ACLR” state: (-11.2 N, *p* = 0.041). No additional significant differences were observed between states in this test.

### Anterior tibial translation

Anterior tibial translation was the reported outcome for the simulated pivot shift test, and anterior drawer at 0° and 30° of flexion tests. Significant differences were found in 2/3 tests: simulated pivot shift (Fig. 4A) and anterior drawer at 0° of flexion (Fig. 4B).

### Anterior tibial translation – simulated pivot shift

In the pivot shift test, the ACLR showed no significant increase in ATT compared to the Native state, while the addition of the segmental meniscus tear, increased anterior translation significantly from both the native state (+ 2.7 mm, *p* < 0.001) and the ACLR state (+ 1.7 mm, *p* = 0.024). The meniscus repair and the MFL augmentation both significantly reduced ATT compared to the segmental meniscus state (-2.0 mm, *p* = 0.004; -2.1 mm, *p* = 0.003, respectively), and neither differed from native or from each other.

### Anterior tibial translation – anterior drawer at 0 degrees of flexion

In the anterior drawer at 0° of flexion test, the ACLR, Segmental meniscus tear, and meniscus repair states all showed significant increases in ATT compares to native (+ 0.7 mm, *p* = 0.006; + 1.0 mm, *p* < 0.001; + 0.7 mm, *p* = 0.004, respectively). Only the MFL augmentation showed no significant difference from intact and showed a significant decrease in ATT compared to the segmental meniscus tear state (-0.7 mm, *p* = 0.009).

### Range of motion

Range of motion (ROM) in degrees was the reported outcome for the external rotation at 0° and 30° of flexion, internal rotation at 0° and 30° of flexion, valgus at 0° and 30° of flexion, and varus at 0° and 30° of flexion tests. Significant ROM differences between states were found in 3/8 tests: Internal rotation at 0° of flexion (Fig. 4C), valgus at 30° of flexion (Fig. 4D) and varus at 30° of flexion (Fig. 4E).

### Range of motion – IR 0

In the internal rotation test, every subsequent state showed significant increases compared to native, with the largest magnitude of increase in the ACLR and Segmental meniscus tear states (+ 1.6°, *p* < 0.001) and the lowest magnitude of increase in the MFL Augmentation state (+ 1.0°, *p* = 0.005). No significant differences were found between any of the repairs.

### Range of motion – valgus at 30 degrees of flexion

In the valgus rotation test, every subsequent state showed significant increases compared to native, with the largest magnitude of increase in the ACLR and Meniscus repair states (+ 0.6°, *p* = 0.018 and *p* = 0.006, respectively) and the lowest magnitude of increase in the Segmental Meniscus tear and MFL Augmentation state (+ 0.5°, *p* = 0.047 and *p* = 0.024, respectively). No significant differences were found between any of the repairs.

### Range of motion – varus at 30 degrees of flexion

In the varus rotation test, the ACLR and Segmental meniscus tear states showed no significant difference from the native state. However, both the meniscus repair state and the MFL augmentation state showed significant reduction in varus rotation compared to the native, ACLR and Segmental meniscus tear states (*p* < 0.05).

## Discussion

The most important finding of this study was that untreated segmental loss PLMR tears may place the ACLR knee at risk for increased laxity due to significantly increased anterior tibial translation in the simulated pivot shift motion (+ 2.7 mm, *p* < 0.001), and that in-situ PLMR repair with and without MFL augmentation restored anterior tibial translation (-2.1 mm, *p* = 0.003 and -2.0 mm, *p* = 0.004, respectively) to values not significantly different from native. Additionally, while segmental loss PLMR tears did not significantly increase ACLR graft force in any test, in-situ PLMR repair significantly decreased ACL graft force in the simulated pivot shift and anterior drawer at 30° flexion tests to below the values seen both in the ACLR state with an intact meniscus, and ACLR state with segmental PLMR loss, which may contribute to protecting the ACL graft. This could potentially be explained by the differing forces seen between intact ACL and ACL reconstructions grafts. MFL imbrication did not significantly change ACL graft force, anterior tibial translation or range of motion values compared to in-situ PLMR repair, indicating no positive benefit for this procedure in terms of ACL graft protection, but no overconstraint beyond native on knee kinematics. The results of this study indicate that repairing PLMR in conjunction with ACLR could decrease a potential modality of ACL failure.

In a biomechanical study investigating contact kinematics after sectioning of the posterior horn of the lateral meniscus and subsequent repair in the ACL intact knee, Schillhammer et al. [21] demonstrated that posterior horn detachment resulted in increased peak contact pressures and decreased average tibial contact area, with repair restoring those values to normal. Forkel et al. [5] assessed the effect of PLMR tears with and without damage to the MFL on contact pressures in the ACL intact knee. These studies reflect the changes seen within the knee joint through outcome measures linked with those from our study. Geeslin et al. [9] evaluated contact kinematics of PLMR tears with and without intact MFLs in the ACLR knee. Similar to the findings reported by Forkel et al., the authors reported PLMR tears with intact MFL did not significantly affect contact kinematics, while PLMR tear with MFL deficiency resulted in significantly decreased lateral compartment contact area and increased contact pressure when compared to the intact state possibly revealing the importance of the given structures and their role in stability of the joint.

﻿ Prior evaluation of segmental loss PLMR tears and in situ repair have been limited to investigation of the restoration of tibiofemoral contact kinematics in the ACL intact knee or the ACL deficient knee [13, 18]. Shybut et al. [23] also evaluated the effect of PLMR tears on the stability of the ACL-deficient knee with simulated Lachman and pivot shift tests, in a protocol which consisted of complete transection of the PLMR and MFLs. The authors reported that PLMR tear in the ACL deficient knee resulted in increased ATT of the lateral compartment during pivot shift test; however, no difference in ATT was observed in the Lachman test when compared with PLMR intact state. These results are similar to the findings observed in the present study, which demonstrated increase ATT with pivot shift testing in the ACLR knee. The PMR repair reducing the forces on the ACLR could largely be due to the underestimated importance of the meniscal root on ACL stability, backed up by the results of this study.

Tang and colleagues [24] recently evaluated the biomechanical effect of PLMR footprint avulsion and repair on the ACLR knee. They demonstrated convincing results that PLMR tear increased ATT of the ACLR knee by approximately 1 mm under anterior tibial loading, and that anatomic repair subsequently reduced this increase in translation. These are similar to our results with segmental loss tears, although our findings showed ATT was most increased with pivot shift testing, and to a greater extent (1.7 mm). The researchers also reported that ACL graft force was paradoxically increased after PLMR repair when compared to the torn root. However, this study did not evaluate the effect of segmental PLMR defect with subsequent in-situ repair, or the effect of meniscofemoral ligament augmentation of the repair. The present study found that in-situ PLMR repair with or without MFL augmentation significantly reduced ACL graft force. Although testing the knee to more extreme limits may elucidate differences between the MFL augmentation states. Further, in situ PLMR was the surgery of choice in this study and exhibited promising results, but additional studies are necessary to pinpoint the ideal technique for PLM repair.

In a retrospective review, Anderson et al. studied 16 patients undergoing PLMR repair or reattachment combined with ACL reconstruction; and at a mean follow-up of 53.6 months, the authors reported no ACL failures and improvements in all patient reported outcome scores showing the efficacy of the surgery of interest in our own study [1]. ﻿In a cohort comparison study, Shelbourne et al. [22] evaluated a cohort of patients with PLMR tears left in situ at the time of ACL reconstruction versus a separate cohort without PLMR tears, with an average follow-up of 10.6 years. The group with tears left in situ had a decrease in lateral compartment joint space of approximately 1 mm relative to the contralateral knee, although no differences in patient reported outcome scores were found between the two cohorts. This highlights what complications could be expected following the presence, or absence, of the surgeries investigated in the present study.

This result suggests that mobilizing the meniscus to the anatomic footprint may not be necessary to restore meniscus function, thus avoiding the morbidity associated with extensive release of the posterior menisco-capsular attachments. However, further research is necessary to directly compare the biomechanics of in-situ repair vs. anatomic fixation. The MFL imbrication did not show any significant differences from the in situ PLMR repair alone in terms of range of motion or ACL graft force and may not be necessary.

The design of the study on the robot necessitated that ACLR and PLMR root repair be performed in an open fashion; although the medial parapatellar arthrotomy was carefully repaired between each testing state. Additionally, joint loads applied by the robot were inferior to in-vivo loads experienced by the knee joint, and higher joint loads may have demonstrated larger differences in graft force. Careful attention was paid to preservation of the meniscofemoral ligament with the segmental meniscal loss state, however there is the possibility that some of the attachments were sacrificed by the nature of the meniscal resection.

## Conclusion

In-situ PLMR repair eliminated pivot shift laxity through ATT and reduced force on the ACL graft, indicating that this procedure may be ACL graft-protective. MFL augmentation was not shown to have any effect on graft force or knee kinematics and untreated PLMR tears may place an ACL graft at higher risk. This study suggests concomitant repair to minimize additional forces on the ACL graft.

## Data Availability

The datasets used and/or analyzed during the current study are available from the corresponding author on reasonable request.

## Abbreviations

PLMR: Posterior lateral meniscal root
MFL: Meniscofemoral ligament
ACL: Anterior cruciate ligament
ACLR: Anterior cruciate ligament reconstruction
IR: Internal rotation
ER: External rotation
ATT: Anterior tibial translation
ROM: Range of motion
